# Developing a manager-informed indicator framework for public sport facility performance measurement: Evidence from perceived indicator importance

**DOI:** 10.1371/journal.pone.0361375

**Published:** 2026-11-13

**Authors:** Xuanye Pan, Caiying Chen, Nor Eeza Zainal Abidin, Mohd Salleh Aman

**Affiliations:** 1 Faculty of Sports and Exercise Science, University of Malaya, Kuala Lumpur, Malaysia; 2 School of Physical Education and Sport Science, Fujian Normal University, Fuzhou, Fujian, China; I-Shou University, TAIWAN

## Abstract

Public sport facility performance measurement draws on provision, user-experience, and distributive indicators, yet these indicators are often considered separately and their roles within a common framework remain unclear. This study developed a manager-informed input–output indicator framework for organising candidate indicators before operational performance measurement. A cross-sectional survey of 228 personnel with management-related responsibilities for public sport facilities in Fujian Province, China, asked participants to rate the importance of five provision-side and eight outcome-side indicators on a five-point scale. The main model retained all 13 indicators and specified both blocks as composites; two reduced-indicator models served as sensitivity analyses. Mean importance ratings ranged from 3.68 to 3.87, and between 62.7% and 70.6% of respondents rated each candidate indicator as important or very important. The Input Importance Composite was positively associated with the Output Importance Composite (β = 0.601, t = 11.879, p < 0.001, 95% confidence interval [0.488, 0.690]). The main model explained 36.2% of the variance in output-side importance ratings, with an effect size of f² = 0.566, and similar associations were observed in the reduced formative–reflective and reduced composite models. Together, the descriptive and structural findings indicate that provision-side and outcome-side importance ratings were positively aligned within participants’ judgments. Supplementary analyses showed no predictive-loss advantage over a linear-model benchmark, and the same-source design left common method concerns unresolved. The observed association is confined to perceived indicator importance; relationships between facility provision and outcomes require separate operational validation. The framework provides a preparatory structure for future validation using administrative, financial, facility, spatial, policy, user, and community data.

## 1. Introduction

Public sport facilities constitute an important form of public service infrastructure. They provide spaces for sport participation, recreation, physical activity, and social interaction. They are also expected to contribute to accessibility, inclusion, and broader public value. As public investment in sport facilities continues, the governance question increasingly extends beyond whether facilities are provided to how their relevance, operation, distribution, and public-facing outcomes should be evaluated. Beyond producing scores or rankings, performance measurement supports accountability, resource allocation, service improvement, and informed public management [1–3].

Research has addressed this evaluation challenge through several established traditions. Efficiency and benchmarking studies have examined utilisation, productivity, cost, and operational performance [4,5]. Service quality and satisfaction studies have focused on users’ experiences of sport and leisure facilities [6,7]. Accessibility and equity research has examined facility availability, spatial distribution, and population-adjusted provision [8–11]. These traditions have generated useful indicators, but they address different evaluative questions and are often applied separately.

The central challenge is how heterogeneous candidate indicators should be organised before they are used in operational performance measurement. Staffing, financial investment, facility number, satisfaction, service quality, per-capita provision, and spatial density may all be relevant, but they represent different components of the evaluation domain. Their inclusion in a single framework requires an explicit account of their conceptual roles; simple aggregation is insufficient. In addition, comparatively limited attention has been paid to the managerial perspective, despite managers’ involvement in planning, resource deployment, facility operation, maintenance, service delivery, and reporting. Managers’ perceived importance ratings complement evidence from users, residents, policymakers, and objective facility data by indicating which candidate indicators are regarded as relevant and operationally interpretable within management practice.

Against this background, the present study develops a manager-informed input–output indicator framework for future public sport facility performance measurement. The empirical analysis is confined to perceived importance ratings provided by public sport facility management personnel for five provision-side and eight outcome-side candidate indicators. Because the candidate indicators are conceptually heterogeneous, the main model specifies the input-side and output-side blocks as composites. Two reduced-indicator models are used as sensitivity specifications to examine whether the principal association remains stable under narrower indicator coverage and alternative measurement logic.

The study makes three contributions. First, it provides a structured approach for organising heterogeneous candidate indicators before operational performance assessment is undertaken. Second, it applies composite measurement logic to distinguish components of an indicator domain from interchangeable manifestations of a single latent construct. Third, it examines whether the association between input-side and output-side importance ratings remains stable across a full composite model and two reduced-indicator sensitivity models. The resulting framework provides a preparatory basis for future research using administrative, facility, financial, spatial, policy, and user-based data.

## 2. Literature review

### 2.1. Public sport facility performance measurement as a multidimensional public service issue

Public sport facilities are expected to serve multiple public purposes. In addition to providing physical opportunities for sport and recreation, they may be evaluated in relation to resource use, service delivery, accessibility, inclusion, community participation, and public value. Performance measurement in this context involves choices about which dimensions of public provision should be made visible and how the resulting information should be used for accountability, management, and improvement [1,2,12].

These multiple purposes require different types of evidence. Facility inventories, staffing records, financial information, and utilisation measures describe aspects of provision and operation. Satisfaction and perceived quality capture users’ evaluations of service experiences. Per-capita and spatial indicators describe the adequacy and distribution of provision. Each indicator family illuminates a different aspect of public sport facility performance, but none provides a complete assessment on its own. The multidimensionality of the field has consequently produced several partially overlapping evaluation traditions.

### 2.2. Existing approaches to public sport facility performance measurement

Previous research can be organised into four broad traditions: efficiency and benchmarking, service quality and satisfaction, accessibility and equity, and composite or multi-criteria assessment. Each tradition contributes a distinct indicator family and addresses a different performance question.

#### 2.2.1. Efficiency and benchmarking approaches.

Efficiency and benchmarking studies examine how facilities use resources and produce measurable operational outputs. Common indicators include utilisation, attendance, operating costs, productivity, financial performance, and facility outputs. Such approaches permit comparison across facilities and can support value-for-money assessment, operational diagnosis, and managerial decision-making [4,5].

The principal contribution of this tradition is to make resource use and operational activity comparable. Its limitation is that efficient operation does not necessarily imply positive user experience, equitable access, or adequate spatial distribution. Efficiency and benchmarking indicators form an important part of public sport facility evaluation, but they address a narrower question than the overall public value of facility provision.

#### 2.2.2. Service quality and satisfaction approaches.

Service quality and satisfaction research focuses on how users experience sport and leisure facilities. Studies of public aquatic and leisure centres have examined relationships among service problems, perceived service quality, benefits, satisfaction, loyalty, and behavioural intentions [6,7]. This tradition demonstrates that facility evaluation should incorporate service delivery and users’ experiences alongside physical provision and financial indicators.

However, satisfaction and service quality measures are shaped by users’ expectations, prior experience, and comparison standards. They primarily represent people who already use facilities and may provide limited information about non-users, unmet demand, or communities facing access barriers. These indicators are valuable for assessing user-facing experience, but they do not independently represent distributive adequacy or the complete performance of a public facility system.

#### 2.2.3. Accessibility and equity approaches.

Accessibility and equity studies examine whether sport facilities are sufficiently available and how opportunities are distributed across populations and places. This research uses indicators such as proximity, population-adjusted provision, service coverage, facility area per capita, spatial density, and accessibility scores. Empirical studies have linked facility proximity and availability with opportunities for sport participation [9]. Recent Chinese research has examined community-level accessibility, spatial allocation, and social or distributive equity [8,10,11].

This tradition contributes evidence that cannot be obtained from internal operations or current-user satisfaction alone. It makes visible whether provision is geographically and demographically adequate. Nevertheless, accessibility studies commonly prioritise spatial diagnosis and do not always specify how distributive indicators should be interpreted alongside staffing, finance, utilisation, service quality, or managerial information.

#### 2.2.4. Composite and multi-criteria approaches.

Composite and multi-criteria approaches attempt to bring several performance dimensions into a broader assessment system. They may include financial, operational, organisational, accessibility, service quality, satisfaction, and public value indicators to support monitoring, comparison, or strategic management [5,12].

The central methodological issue is not simply the number of indicators included. Composite assessment also requires decisions about indicator selection, grouping, weighting, aggregation, and interpretation. Research on national sport-performance rankings similarly shows that comparative results depend on indicator coverage and the transparency of the measurement and weighting rules applied [13]. Without an explicit organising logic, a composite score may conceal how performance has been defined.

Related issues arise in other public-service facilities. Moldovan and Moldovan [14], for example, incorporated community involvement and development into the evaluation of a healthcare facility, illustrating that facility assessment may extend beyond conventional satisfaction, efficiency, or operational indicators. Across these contexts, the usefulness of a composite framework depends on whether the conceptual roles of its indicators are specified before aggregation.

### 2.3. The relevance of the managerial perspective

Performance indicators must be conceptually defensible, but they must also be interpretable within the organisations expected to collect and use them. Public-sector performance information is embedded in planning, budgeting, reporting, accountability, and operational control; it is not merely an academic measurement exercise [1,15,16]. Within sport organisations and facilities, managers similarly translate policy expectations into decisions about staffing, maintenance, resource allocation, programming, service delivery, and routine monitoring [12,17].

Managers are relevant informants for assessing the perceived importance and operational interpretability of candidate indicators. Their judgments can show which types of information they regard as meaningful for future facility evaluation and which indicators are likely to be interpretable within management and reporting practices. This form of evidence is particularly relevant at the preparatory stage of framework development, before candidate indicators are operationalised using observed facility and outcome data.

The managerial perspective contributes one organisational layer within a broader multi-stakeholder evaluation. Managers provide insight into the operational relevance and reporting interpretability of candidate indicators. Evidence from users, residents, and policymakers, together with administrative and spatial data, can provide complementary information on service experience, unmet demand, strategic and equity objectives, and provision conditions.

### 2.4. Measurement logic: distinguishing provision-side from outcome-side indicators

The indicator traditions reviewed above generate conceptually different forms of evidence. Provision-side indicators describe resources, organisational arrangements, and the physical scale of facility supply, including staffing, instructor support, financial investment, facility number, and facility area. Outcome-side indicators describe domains relevant to what public sport facilities are expected to support or deliver, including satisfaction, perceived quality, population-adjusted provision, and spatial adequacy. The distinction concerns the role of an indicator within the proposed framework, not whether an indicator is inherently an input or output in every possible evaluation system.

This distinction also requires an appropriate measurement specification. Reflective measurement assumes that indicators are manifestations of a common underlying construct and are expected to covary because they share that common source. Removing one reflective indicator does not ordinarily redefine the underlying construct. Composite and formative specifications both use an indicator-to-construct direction, but they are not conceptually identical. In a composite specification, the construct is defined as a weighted combination of its indicators. In a formative specification, the indicators are treated as contributing causes of an underlying construct. In both cases, the indicators may represent heterogeneous and non-interchangeable components, and removing an indicator may alter the content represented. Model specification, therefore, should follow the conceptual relationship between the indicators and the construct rather than statistical convention alone [18–21]. A recent applied study provides a context-specific illustration of this principle [22].

The candidate indicators in the present study conform more closely to composite logic. Administration staff, instructors, investment, facility number, and facility area represent distinct components of provision; they are not interchangeable expressions of a single latent characteristic. The output-side domain is similarly heterogeneous: satisfaction and perceived quality represent experiential considerations, whereas per-capita provision and area density represent distributive or spatial considerations. These indicators may jointly define a broader outcome-oriented composite, but they should not be assumed to be interchangeable manifestations of one homogeneous latent construct.

Comparable measurement problems have been addressed through formative index construction in service and hospitality research, where distinct dimensions jointly define a multidimensional domain [23,24]. On this basis, the present study specifies both full indicator blocks as composites in the main model. A reduced reflective output specification is retained only as a sensitivity model, permitting an empirical comparison without treating the narrower reflective structure as the conceptual definition of the full outcome-side domain.

### 2.5. Integrated gap and framework response

Collectively, the literature reveals three connected limitations. First, efficiency, user-experience, accessibility, and composite traditions have produced relevant indicator families, but they remain only partially integrated. Recent conceptual work similarly identifies fragmentation across public sport facility evaluation traditions and the need to connect operational, user-facing, distributive, and governance considerations more systematically [3]. Second, the managerial perspective has been used less frequently as evidence for the preparatory organisation of candidate indicators. Third, studies that combine multiple indicators do not always make the assumed relationship between the indicators and the broader measurement domain explicit.

These limitations create a need for a framework that precedes operational performance calculation. Such a framework should distinguish provision-side from outcome-side indicator roles, retain the multidimensional content of the candidate domain, and identify how relevant indicators are perceived by those responsible for facility management and reporting. The present study addresses this preparatory stage by organising these ratings into a manager-informed input–output indicator framework and examining the framework using composite measurement logic. The resulting structure can subsequently be tested using administrative, financial, spatial, facility, policy, and user-based evidence.

### 2.6. Conceptual definitions and analytical expectation

#### 2.6.1. Provision-side indicators as the input importance composite.

Provision-side indicators are candidate measures of the resources, organisational arrangements, and physical conditions associated with public sport facility provision. The present study includes administration staff, social sports instructors, public financial investment, facility number, and facility area. The survey captures participants’ ratings of how important these indicators should be for future measurement; it does not record the observed level of each resource. The resulting composite is termed the Input Importance Composite. “Input” denotes the indicators’ position in the framework, while “importance” identifies the empirical quantity measured.

#### 2.6.2. Outcome-side indicators as the output importance composite.

Outcome-side indicators are candidate measures of domains that public sport facilities may be expected to support or deliver. They include satisfaction, core product quality, service quality, social network quality, relationship investment quality, facility number per capita, facility area per capita, and facility area density. The survey measures participants’ perceived importance of these indicators and does not measure actual facility outcomes. The indicators form the Output Importance Composite. The composite combines experiential and distributive components without assuming that they are interchangeable manifestations of a single latent outcome.

#### 2.6.3. Analytical expectation: Empirical coherence of the proposed framework.

The analytical expectation is that greater importance assigned to provision-side candidate indicators will be accompanied by greater importance assigned to outcome-side indicators. A positive association between the two composites would therefore indicate coherence in the observed rating patterns rather than an operational input–output relationship.

## 3. Methods

### 3.1. Study design and analytical scope

This study used a cross-sectional survey of public sport facility management personnel to develop and assess a manager-informed input–output indicator framework for future public sport facility performance measurement. The empirical scope was limited to these importance ratings; no operational facility, service-delivery, efficiency, or user-outcome data were included.

The analytical objective was to examine whether these ratings could be organised into a coherent input–output indicator framework. Input-side indicators represented provision-related candidate measures relevant to future operational assessment, and output-side indicators represented candidate domains associated with user-facing experience and distributive adequacy. The structural path quantified the association between the two rating composites.

### 3.2. Participants, sampling, recruitment, eligibility screening, and ethics

The target population comprised personnel with management-related responsibilities for public sport facilities in Fujian Province. Participants were recruited purposively through an established professional working network originating from the Fujian Province sport facility statistical survey conducted in 2019. The network was established for sport facility statistical data collection and coordination and included personnel from sport administrative departments, subdistrict and community organisations, facility-operating enterprises, universities, and other organisations involved in sport facility-related work.

Before survey invitations were distributed, potential participants were identified within this network on the basis of their known occupational or assigned responsibilities related to public sport facility administration, operation, coordination, statistical reporting, or other management-related duties. Recruitment invitations were directed to these individuals rather than to all members of the network.

Recruitment and data collection were conducted from 25 July to 5 August 2024 using Wen Juan Xing, an online questionnaire administration platform. The platform was used to administer the questionnaire and implement the programmed consent and eligibility-screening procedures; it was not used as the sampling frame. The study protocol and questionnaire were approved by the University of Malaya Research Ethics Committee under reference number UM.TNC 2/UMREC_2352.

After providing informed consent, respondents were required to answer the mandatory eligibility-screening question: “Do you have any experience in the development, management, or research of sports facilities?” Respondents selecting “No” were automatically directed to the end of the survey. This question served as an additional screen for relevant sport-facility experience rather than as the sole criterion for establishing management-related responsibilities, which had been identified before recruitment through the professional working network and participants’ known roles. Of 269 individuals who accessed the survey, one did not provide consent and 40 were screened out by the eligibility question. The remaining 228 participants completed all substantive items and constituted the final analytic sample.

### 3.3. Candidate indicators and survey instrument

The questionnaire measured the perceived importance of candidate indicators for inclusion in a future public sport facility performance measurement framework. Indicators were drawn from prior research on sport facility provision, public sport service evaluation, operational efficiency, service and experience quality, user satisfaction, and accessibility. The instrument contained two substantive indicator blocks.

The input-side block comprised five provision-related candidate indicators: administration staff, social sports instructors, public financial investment in public sport facilities, number of public sport facilities, and public sport facility area. The output-side block comprised eight outcome-oriented candidate indicators: satisfaction with the use of public sport facilities, core product quality, service quality, social network quality, relationship investment quality, number of public sport facilities per capita, public sport facility area per capita, and public sport facility area density.

Respondents rated how important each candidate indicator was for inclusion in a public sport facility performance measurement system. All 13 substantive items used a 5-point Likert-type importance scale ranging from 1 = very unimportant to 5 = very important. The resulting responses represented perceived indicator importance. The candidate indicators, conceptual meanings, and source bases are summarised in Table 1. The questionnaire items are provided in S1 Appendix, and the corresponding variable definitions and numerical coding are provided in S1 Data.

**Table 1:** Candidate indicators used in the manager-informed input–output indicator framework.

Construct block	Code	Candidate indicator	Conceptual meaning	Source basis
Input-side block	Input 1	Administration staff	Administrative and coordination capacity for facility management	[25,26]
Input-side block	Input 2	Social sports instructors	Service-delivery and programme support capacity	[26,27]
Input-side block	Input 3	Public financial investment	Fiscal support for construction, maintenance, and service provision	[26,28]
Input-side block	Input 4	Number of public sport facilities	Scale of facility provision	[26,27]
Input-side block	Input 5	Public sport facility area	Spatial and physical capacity of provision	[26,27]
Output-side block	Output 1	Satisfaction with the use of public sport facilities	Overall user-centred evaluative outcome	[29,30]
Output-side block	Output 2	Core product quality	Quality of the core facility and sport offering	[31]
Output-side block	Output 3	Service quality	Quality of service delivery and support	[31]
Output-side block	Output 4	Social network quality	Quality of social atmosphere and interaction	[31]
Output-side block	Output 5	Relationship investment quality	Quality of relationship-building and sustained user orientation	[31]
Output-side block	Output 6	Number of public sport facilities per capita	Facility availability relative to population	[26,32]
Output-side block	Output 7	Public sport facility area per capita	Facility space available relative to population	[26,32]
Output-side block	Output 8	Public sport facility area density	Spatial intensity of facility provision	[27,32]

Note. The survey measured participants’ perceived importance ratings of candidate indicators for future public sport facility performance measurement. Responses were recorded on a 5-point importance scale ranging from 1 = very unimportant to 5 = very important.

### 3.4. Data screening, missing data, sample-size rationale, and descriptive analysis

Following the consent and eligibility-screening procedures described in Section 3.2, the analytic dataset comprised 228 eligible participants. The 40 individuals who did not meet the mandatory eligibility criterion were screened out before accessing the substantive indicator-importance items and contributed no substantive data to the analytic dataset. All 228 eligible respondents completed the 13 indicator-importance items. Accordingly, there were no item-level missing data, and no data imputation was performed.

A power-based sample adequacy check was conducted for the primary structural association between the Input Importance Composite and the Output Importance Composite using G*Power version 3.1.9.7 [33]. The calculation used the F-test procedure for linear multiple regression, fixed model, R² deviation from zero, with one predictor. Assuming Cohen’s medium effect-size benchmark of f² = 0.15 [34], an alpha level of 0.05, and statistical power of 0.80, the required minimum sample size was 55 cases, with an actual power of 0.805. The final analytic sample of 228 participants exceeded this power-based benchmark. This calculation assessed sample adequacy for detecting the single primary structural association; it did not establish the representativeness of the purposively recruited sample or the precision of all measurement estimates. Estimate precision was assessed separately using percentile bootstrap confidence intervals based on 5,000 subsamples.

Respondent characteristics were summarised using frequencies and percentages for categorical variables. For each of the 13 candidate indicators, the mean, standard deviation, and number and percentage of respondents selecting ratings of 4 or 5 were calculated. These statistics described the absolute level and dispersion of item ratings before model estimation.

Descriptive item statistics were interpreted separately from the outer weights estimated in the composite models. Item means represented the average absolute importance assigned to an indicator, whereas outer weights represented its unique contribution to the relevant composite conditional on the other indicators in the same block. A comparatively large outer weight was not interpreted as evidence that an indicator had received the highest absolute importance rating.

### 3.5. Measurement specification and model strategy

The analysis followed a composite indicator logic. The full input-side and output-side blocks comprised conceptually heterogeneous components of an indicator framework; they were not treated as interchangeable manifestations of a single latent trait. This specification followed established guidance that the conceptual relationship between indicators and the domain they define—not empirical fit alone—should determine the measurement specification [18–21].

Three models were estimated. Model A was the main model and retained the full surveyed candidate indicator set. All five input-side indicators formed the Input Importance Composite, and all eight output-side indicators formed the Output Importance Composite. Both blocks were specified as composites.

Models B and C used a reduced indicator set carried forward from the preliminary refinement of the formative–reflective specification. Because the two blocks had different measurement roles in that preliminary specification, indicator reduction was not based on a single common statistical rule. For the formative input-side block, Input 2 and Input 3 showed limited unique contributions relative to the retained input indicators and were excluded from the reduced set. For the reflective output-side block, Output 4 showed a comparatively weaker association with the proposed unidimensional output construct and was excluded. The resulting reduced set comprised Input 1, Input 4, Input 5, Output 1, Output 2, Output 3, Output 5, Output 6, Output 7, and Output 8.

These exclusions defined the reduced sensitivity specifications only. Model A retained all 13 candidate indicators to preserve the content coverage of the surveyed indicator set. Models B and C examined whether the primary structural association remained stable when the reduced indicator set was applied under alternative measurement specifications.

Model B was a reduced-indicator formative–reflective sensitivity model. Its input-side block was specified formatively, and its output-side block was specified reflectively. Model C used the same reduced indicator set but specified both blocks as composites. Comparing Models B and C held the reduced indicator set constant while varying the measurement specification of the output-side block. The three model specifications are shown in Fig 1.

**Fig 1:**
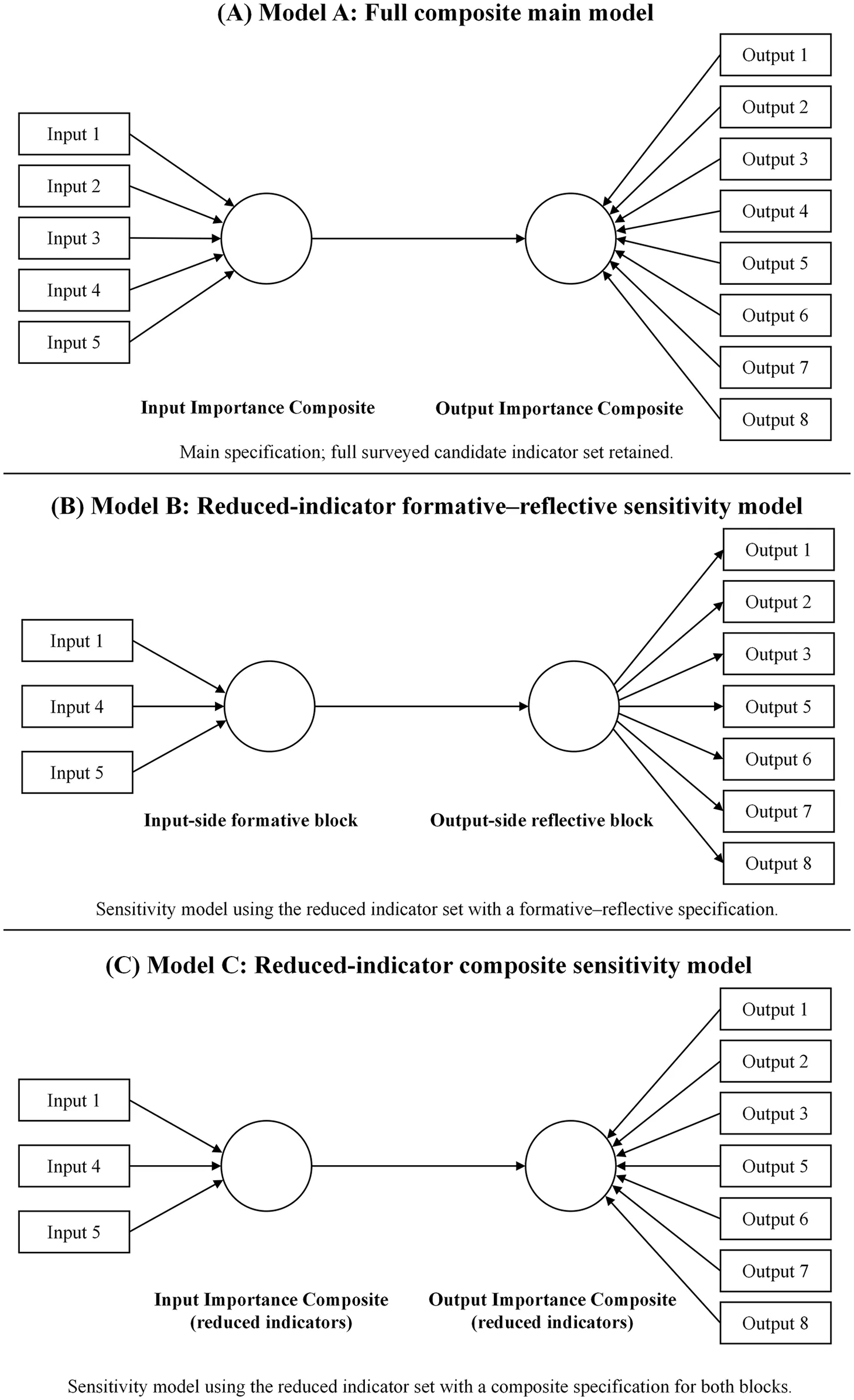
Alternative model specifications used in the analysis.

### 3.6. PLS composite modelling procedure

The models were estimated using SmartPLS 4, version 4.1.1.6. Partial least squares structural equation modelling was used because the analysis included composite models, a formative measurement specification, and estimation of the structural association between the indicator blocks. PLS-SEM supports the estimation of composite and formative models and is suitable for analyses emphasising indicator weights, explained variance, and structural relationships [19,21].

For Models A and C, both indicator blocks were specified as composites and estimated using outer weighting Mode B. In Model B, Mode B was used for the formative input-side block and Mode A for the reflective output-side block. The PLS algorithm used the path weighting scheme, a maximum of 300 iterations, and a stop criterion of 1.0 × 10^−7^.

The composite measurement assessment for Models A and C examined indicator collinearity, outer weights, and supplementary outer loadings. The formative input-side block in Model B was assessed using the same indicator-level criteria [18,19,21]. Collinearity was assessed using variance inflation factors. Outer weights represented each indicator’s unique contribution to the corresponding block conditional on the other indicators, whereas outer loadings were reported as supplementary evidence of absolute indicator relevance. Candidate indicators in Model A were not removed solely on the basis of nonsignificant outer weights; their retention also reflected the conceptual coverage of the surveyed candidate indicator set. Models B and C retained the prespecified reduced indicator set described in Section 3.5.

For the reflective output-side construct in Model B, measurement assessment included outer loadings, Cronbach’s alpha, ρ_A, composite reliability, and average variance extracted. Discriminant validity assessment using the heterotrait–monotrait ratio and cross-construct cross-loading comparisons was not applicable because Model B contained only one reflectively specified construct.

Redundancy analysis was not conducted because the survey did not include an independent global criterion or alternative measure representing the corresponding formative or composite domains. Such an alternative measure is required for redundancy analysis as an assessment of convergent validity [19]. No post hoc criterion was constructed from the same indicators.

Statistical inference was based on bootstrapping with 5,000 subsamples, two-tailed tests, and a 5% significance level. Percentile bootstrap 95% confidence intervals were reported for outer weights and structural path coefficients. For Models B and C, indicator-level percentile bootstrap 95% confidence intervals were additionally reported for outer loadings. The stability of outer weights was interpreted primarily according to whether the corresponding confidence interval excluded zero, with two-tailed p values reported as complementary evidence. For the reflective output-side construct in Model B, the significance and confidence intervals of the outer loadings were also examined.

### 3.7. Structural and supplementary predictive assessment

The structural association from the Input Importance Composite to the Output Importance Composite was assessed using the path coefficient, bootstrapped t value, two-tailed p value, and 95% percentile bootstrap confidence interval. Explanatory power was assessed using R² and adjusted R², and the effect size of the structural relationship was assessed using f². The path quantified the association between participants’ input-side and output-side importance ratings.

A supplementary predictive assessment was conducted for the main Model A using PLSpredict with 10 folds and 10 repetitions. At the indicator level, Q²predict, root mean square error (RMSE), and mean absolute error (MAE) were obtained for each output indicator and compared with the corresponding linear-model benchmark [35]. Construct-level Q²predict, RMSE, and MAE were also reported for the Output Importance Composite. Predictive loss was further compared with the linear-model benchmark using the cross-validated predictive ability test (CVPAT) at both the indicator and construct levels [36].

Sensitivity analysis compared the direction and magnitude of the structural association across Model A, the full composite main model; Model B, the reduced-indicator formative–reflective model; and Model C, the reduced-indicator composite model. The sensitivity analysis was used to determine whether the principal association remained comparable across the full and reduced indicator sets and the alternative measurement specifications.

### 3.8. Common method variance assessment

Because all substantive indicators were rated by the same respondents using the same Likert-type importance scale, common method variance was considered as a potential source of bias. Procedural remedies included voluntary participation, anonymous survey completion, neutral wording of the indicator-importance items, and questions framed around indicator inclusion, not respondents’ evaluations of their own facility performance. The questionnaire was pilot-tested before the main survey to improve clarity and reduce ambiguity, and demographic questions were separated from the substantive indicator-rating items.

Sampling adequacy and factorability were examined using the Kaiser–Meyer–Olkin measure and Bartlett’s test of sphericity. All 13 substantive indicator-importance items were then entered into an unrotated exploratory factor analysis, and the percentage of variance explained by the first unrotated factor was examined. Because this diagnostic cannot establish whether common method variance materially biased the model estimates, it was used only as a cautionary assessment and not as evidence that common method bias had been eliminated [37].

### 3.9. Data anonymisation and availability

The anonymised survey dataset, including a codebook defining the variables and numerical category codes, is provided as S1 Data. The questionnaire items are provided as S1 Appendix.

## 4. Results

### 4.1. Sample characteristics and descriptive indicator-importance ratings

The analytic sample comprised 228 public sport facility management personnel. Respondents were affiliated with public institutions (34.6%), enterprises (33.8%), government departments (22.4%), and research institutions (9.2%). Sample characteristics are presented in Table 2.

**Table 2:** Characteristics of the public sport facility management personnel included in the analytic sample (N = 228).

Characteristic	Category	n	%
Gender	Male	116	50.9
	Female	112	49.1
Age, years	18–29	68	29.8
	30–39	80	35.1
	40–49	51	22.4
	50–59	27	11.8
	≥60	2	0.9
Education level	Lower secondary education or below	2	0.9
	Upper secondary or technical secondary education	7	3.1
	Junior college / college diploma	51	22.4
	Bachelor’s degree	84	36.8
	Postgraduate degree or above	84	36.8
Organisation type	Government department	51	22.4
	Public institution	79	34.6
	Research institution	21	9.2
	Enterprise	77	33.8

Note. Values are presented as n and percentages. Percentages were calculated using the final analytic sample of 228 respondents.

Participants’ mean importance ratings for the 13 candidate indicators ranged from 3.68 to 3.87. Public financial investment in public sport facilities (Input 3; M = 3.87, SD = 1.25) and public sport facility area density (Output 8; M = 3.86, SD = 1.22) received the highest average ratings. Both indicators were rated as important or very important by 161 respondents (70.6%). Social sports instructors (Input 2) had the lowest proportion of ratings of 4 or 5, with 143 respondents (62.7%; M = 3.71, SD = 1.17). Descriptive statistics for all 13 candidate indicators are presented in Table 3.

**Table 3:** Descriptive statistics for participants’ perceived importance ratings of candidate indicators.

Indicator	Mean	SD	Rated 4 or 5, n (%)
Input 1	3.68	1.26	147 (64.5)
Input 2	3.71	1.17	143 (62.7)
Input 3	3.87	1.25	161 (70.6)
Input 4	3.82	1.25	156 (68.4)
Input 5	3.75	1.31	154 (67.5)
Output 1	3.80	1.27	158 (69.3)
Output 2	3.81	1.32	158 (69.3)
Output 3	3.77	1.29	157 (68.9)
Output 4	3.68	1.29	146 (64.0)
Output 5	3.70	1.28	150 (65.8)
Output 6	3.77	1.27	147 (64.5)
Output 7	3.71	1.30	146 (64.0)
Output 8	3.86	1.22	161 (70.6)

Note. Ratings were recorded on a 5-point importance scale ranging from 1 = very unimportant to 5 = very important. “Rated 4 or 5” indicates the number and percentage of respondents selecting important or very important. Percentages were based on 228 complete responses for each item.

### 4.2. Common method variance diagnostic

The Kaiser–Meyer–Olkin value was 0.928, and Bartlett’s test of sphericity was significant, χ²(78) = 1705.233, p < 0.001. The first unrotated factor accounted for 51.178% of the total variance across the 13 indicator-importance items. This diagnostic did not rule out common method concerns. Subsequent model estimates were interpreted as same-source patterns in indicator importance.

### 4.3. Main model: Full composite specification

Model A retained all five input-side and eight output-side candidate indicators, with both blocks specified as composites. The composite measurement and structural results are reported below.

#### 4.3.1. Composite measurement assessment.

For the Input Importance Composite, VIF values ranged from 1.781 to 2.652. For the Output Importance Composite, VIF values ranged from 1.863 to 2.460. No indicator VIF exceeded 3.0.

Table 4 presents the outer weights, supplementary outer loadings, and collinearity statistics for the Input Importance Composite. Number of public sport facilities had the largest estimated outer weight (weight = 0.491, p = 0.004, 95% CI [0.133, 0.795]). Public sport facility area had the second-largest estimated weight (weight = 0.399), but its confidence interval included zero. Administration staff had a positive estimated weight. The estimated weights for social sports instructors and public financial investment were close to zero. All five indicators had positive outer loadings, ranging from 0.649 to 0.921.

**Table 4:** Composite measurement results for the Input Importance Composite.

Indicator	Outer weight	p	95% CI	Outer loading	VIF
Input 1: Administration staff	0.220	0.193	[-0.115, 0.546]	0.805	2.175
Input 2: Social sports instructors	−0.014	0.923	[-0.280, 0.312]	0.649	1.781
Input 3: Public financial investment	0.029	0.883	[-0.341, 0.435]	0.730	2.111
Input 4: Number of public sport facilities	0.491	0.004	[0.133, 0.795]	0.921	2.428
Input 5: Public sport facility area	0.399	0.055	[-0.052, 0.765]	0.899	2.652

Note. CI = percentile bootstrap 95% confidence interval. The input-side block was specified as a composite. Outer weights indicate unique contributions to the composite; outer loadings provide supplementary evidence of indicator relevance. VIF values ranged from 1.781 to 2.652.

The ordering of the outer weights differed from the descriptive item means. Input 3 had the highest mean importance rating, whereas Input 4 had the largest estimated outer weight.

Table 5 presents the results for the Output Importance Composite. Number of public sport facilities per capita had the largest estimated outer weight (weight = 0.299). However, its percentile bootstrap 95% confidence interval included zero, and the weight was not interpreted as a statistically robust unique contribution. Public sport facility area per capita had the second-largest estimated weight, but its confidence interval also included zero. Overall satisfaction, core product quality, and service quality had positive estimated weights. Smaller estimated weights were observed for social network quality, relationship investment quality, and public sport facility area density. All eight indicators had positive supplementary outer loadings, ranging from 0.586 to 0.829.

**Table 5:** Composite measurement results for the Output Importance Composite.

Indicator	Outer weight	p	95% CI	Outer loading	VIF
Output 1: Overall satisfaction	0.230	0.192	[-0.138, 0.557]	0.816	2.289
Output 2: Core product quality	0.183	0.232	[-0.112, 0.494]	0.786	2.109
Output 3: Service quality	0.192	0.169	[-0.077, 0.478]	0.766	2.006
Output 4: Social network quality	−0.065	0.652	[-0.335, 0.234]	0.586	1.863
Output 5: Relationship investment quality	0.049	0.757	[-0.272, 0.358]	0.755	2.460
Output 6: Number of public sport facilities per capita	0.299	0.041	[-0.020, 0.558]	0.825	2.093
Output 7: Public sport facility area per capita	0.261	0.111	[-0.066, 0.576]	0.829	2.280
Output 8: Public sport facility area density	0.081	0.624	[-0.266, 0.388]	0.721	2.095

Note. CI = percentile bootstrap 95% confidence interval. The output-side block was specified as a composite. Outer weights indicate unique contributions to the composite; outer loadings provide supplementary evidence of indicator relevance. VIF values ranged from 1.863 to 2.460.

The candidate indicators did not consistently show statistically robust unique weights. Input 4 was the only indicator whose outer-weight confidence interval excluded zero; all output-side outer-weight confidence intervals included zero. Nevertheless, all indicators showed positive supplementary outer loadings. All surveyed candidate indicators were retained in Model A.

#### 4.3.2. Structural association between the input importance composite and the output importance composite.

The structural path from the Input Importance Composite to the Output Importance Composite was positive and statistically significant, β = 0.601, t = 11.879, p < 0.001, 95% CI [0.488, 0.690]. Model A explained 36.2% of the variance in the Output Importance Composite (R² = 0.362, adjusted R² = 0.359), with f² = 0.566.

The coefficient indicates that higher input-side importance ratings were associated with higher output-side importance ratings. Full structural results for Model A are presented in Table 6.

**Table 6:** Structural results for the main model.

Relationship	β	t	p	95% CI	R²	Adj. R²	f²
Input Importance Composite → Output Importance Composite	0.601	11.879	< 0.001	[0.488, 0.690]	0.362	0.359	0.566

Note. CI = percentile bootstrap 95% confidence interval. The structural path represents an association between participants’ perceived importance ratings of input-side and output-side candidate indicators.

#### 4.3.3. Supplementary predictive assessment.

The supplementary predictive assessment for Model A showed positive Q²predict values for all eight output indicators, ranging from 0.097 to 0.214. Relative to the linear-model benchmark, PLS-SEM produced lower RMSE values for five of the eight indicators and lower MAE values for five of the eight indicators, although the pattern was not consistent across indicators. Indicator-level CVPAT comparisons likewise showed no statistically significant predictive-loss advantage over the linear-model benchmark for any of the eight output indicators (all p > 0.05).

At the construct level, the Output Importance Composite yielded Q²predict = 0.260, RMSE = 0.866, and MAE = 0.682. The construct-level CVPAT comparison produced identical rounded predictive losses for PLS-SEM and the linear model (1.355 vs. 1.355), with an average loss difference of 0.000 (t = 0.064, p = 0.949). These results indicate positive predictive relevance, but no consistent predictive-loss advantage over the linear-model benchmark. Detailed results are presented in Table 7.

**Table 7:** Predictive assessment for Model A.

Panel A. Indicator-level PLSpredict results								
**Indicator**	**Q²predict**	**PLS RMSE**	**LM RMSE**	**PLS MAE**	**LM MAE**			
Output 1	0.205	1.138	1.130	0.885	0.888			
Output 2	0.187	1.192	1.190	0.944	0.943			
Output 3	0.181	1.171	1.174	0.918	0.921			
Output 4	0.097	1.232	1.226	0.991	0.992			
Output 5	0.176	1.165	1.169	0.911	0.916			
Output 6	0.210	1.133	1.134	0.906	0.903			
Output 7	0.214	1.151	1.156	0.917	0.918			
Output 8	0.154	1.129	1.130	0.868	0.864			
**Panel B. Construct-level PLSpredict and CVPAT results**								
**Endogenous construct**	**Q²predict**	**RMSE**	**MAE**	**PLS loss**	**LM loss**	**Difference**	**t**	**p**
Output Importance Composite	0.260	0.866	0.682	1.355	1.355	0.000	0.064	0.949

Note. LM = linear-model benchmark. PLSpredict used 10 folds and 10 repetitions. Positive Q²predict values indicate predictive relevance. Indicator-level PLS-SEM prediction errors were compared with the corresponding linear-model errors. Construct-level predictive loss was compared with the linear-model benchmark using CVPAT.

### 4.4. Sensitivity analyses

Models B and C used the reduced indicator set described in Section 3.5. The indicators were not reselected on the basis of the Model A results. Model B specified the input-side block formatively and the output-side block reflectively. Model C specified both reduced blocks as composites. Detailed indicator-level measurement results for both sensitivity models are provided in S1 Table.

For the formative input-side block in Model B, VIF values ranged from 1.977 to 2.308. The outer-weight confidence intervals excluded zero for Input 4 and Input 5 but included zero for Input 1. All three indicators showed positive supplementary outer loadings ranging from 0.827 to 0.917, with corresponding 95% confidence intervals excluding zero. The seven reflective output indicators in Model B had outer loadings ranging from 0.775 to 0.814. All loadings were statistically significant (all p < 0.001), and their percentile bootstrap 95% confidence intervals excluded zero. The reflective output-side construct had Cronbach’s α = 0.903, ρ_A = 0.904, composite reliability = 0.924, and AVE = 0.633.

For Model C, VIF values ranged from 1.977 to 2.308 for the reduced input-side composite and from 2.003 to 2.333 for the reduced output-side composite. The outer-weight confidence intervals excluded zero for Input 4, Input 5, and Output 6, whereas the remaining outer-weight confidence intervals included zero. All indicators showed positive supplementary outer loadings, ranging from 0.811 to 0.923 for the input-side composite and from 0.725 to 0.828 for the output-side composite; all corresponding 95% confidence intervals excluded zero.

The estimated structural coefficients were similar across the three specifications. Model A produced β = 0.601 and R² = 0.362; Model B produced β = 0.595 and R² = 0.354; and Model C produced β = 0.601 and R² = 0.361. Full structural results are presented in Table 8.

**Table 8:** Comparison of Model A, Model B, and Model C.

Model	Indicator set	Measurement specification	β	t	p	95% CI	R²	Adj. R²	f²
Model A	Full 5 input + 8 output indicators	Composite → Composite	0.601	11.879	< 0.001	[0.488, 0.690]	0.362	0.359	0.566
Model B	Reduced indicators	Formative → Reflective	0.595	11.435	< 0.001	[0.475, 0.684]	0.354	0.351	0.549
Model C	Reduced indicators	Composite → Composite	0.601	12.084	< 0.001	[0.519, 0.715]	0.361	0.358	0.564

Note. Model A was the main model and retained the full surveyed candidate indicator set. Models B and C used the reduced indicator set described in Section 3.5. Model B was a reduced-indicator formative–reflective sensitivity model. Model C used the same reduced indicator set as Model B but specified both blocks as composites. All paths represent associations between participants’ perceived importance ratings.

## 5. Discussion

### 5.1. Interpreting coherence in participants’ indicator-importance ratings

The main finding was a positive association between the two importance composites, alongside relatively high ratings across both indicator domains. This pattern supports coherence between provision-side and outcome-side priorities within the observed ratings.

An input–output perspective provides a useful organising logic because the two sides represent different but connected components of future performance measurement. The input side encompasses organisational, fiscal, service-support, and physical conditions of provision, whereas the output side encompasses user-experience and distributive criteria against which that provision may subsequently be evaluated.

The association pertains specifically to perceived indicator importance; relationships between observed provision conditions and operational outcomes require subsequent testing. Model A explained 36.2% of the variation in output-side importance ratings. The remaining variation may reflect organisational and contextual factors not represented in the model.

### 5.2. Contribution relative to existing evaluation traditions

Existing evaluation traditions have generated distinct evidence on resource use, user experience, and the spatial and population distribution of facility opportunities [4,7,8,29]. The present study advances this literature by specifying how provision-related, experiential, and distributive indicators can be positioned within a shared manager-informed input–output framework. Provision-side indicators represent conditions associated with facility supply and operation; outcome-side indicators represent experiential and distributive criteria for evaluating the relevance and adequacy of that provision. Clarifying these conceptual roles before operationalisation provides a basis for subsequent decisions concerning data sources, indicator direction, normalisation, weighting, aggregation, and comparison.

The managerial perspective adds an organisational dimension to this contribution. Performance information is collected, interpreted, reported, and used within organisational settings, and its relevance is shaped by management purposes and practices [16]. Public sport facility management personnel occupy a practical position between policy objectives and facility-level implementation. Their importance ratings indicate which candidate indicators are meaningful within management and reporting contexts.

Within the broader multi-level evaluation agenda identified by Pan et al. [3], the present study develops the manager-informed component of indicator framework design. A multi-source performance measurement system can combine this organisational perspective with evidence from users, residents, policymakers, administrative records, financial data, facility inventories, and spatial analysis. The present framework establishes a structure through which these different forms of evidence may subsequently be connected.

### 5.3. Composite specification and indicator-domain coverage

The measurement contribution of the study lies in aligning the statistical specification with the conceptual status of the candidate indicators. The provision-side indicators represent distinct organisational, fiscal, service-support, and physical components. The outcome-side indicators are similarly heterogeneous: satisfaction and perceived quality represent experiential criteria, whereas per-capita provision and area density represent distributive criteria. Specifying the full blocks as composites preserves this multidimensional content without requiring the indicators to operate as interchangeable manifestations of a single latent characteristic.

The three-model strategy separated conceptual coverage from sensitivity assessment. Model A retained the full surveyed candidate indicator set. Models B and C used the reduced set derived from the preliminary refinement of the formative–reflective specification. This distinction is important because indicators in formative and composite measurement contribute to the content represented by the broader domain. Removing an indicator may narrow or alter that domain even when the resulting model is more parsimonious [18–20].

Social sports instructors illustrate the value of retaining conceptually distinct content. Input 2 made a limited unique statistical contribution in the preliminary formative assessment, but it represents programme delivery, exercise guidance, activity organisation, and participation support. These functions are not captured by administration staff, facility number, or facility area. Recent evidence from China associates social sports instructors with the frequency and duration of residents’ sport participation and emphasises their role in activity organisation and professional guidance [38]. Including Input 2 in Model A preserves the service-support component of public sport facility provision.

Public financial investment demonstrates a different relationship between descriptive importance and conditional statistical contribution. Participants assigned high absolute importance to Input 3. Its unique outer weight was limited after the other provision-side indicators were considered simultaneously. The two statistics address different questions: the mean reflects the absolute importance assigned to the indicator; the outer weight reflects its unique contribution conditional on the remaining indicators in the block. Government sports expenditure may operate as one element within a broader configuration that also includes facility, technological, institutional, and policy conditions [39]. Consequently, Input 3 represents the financial support component of the provision-side domain even though its conditional contribution was small in the present model.

Social network quality highlights the limitations of imposing a homogeneous reflective interpretation on the outcome-side domain. Its comparatively weak association with the reduced reflective output construct is consistent with the interpretation of social and community value as a distinct component of sport experience. This component is conceptually separate from satisfaction, service quality, and distributive adequacy. Yoshida [31] conceptualised social network quality as a separate dimension of sport experience, and subsequent research has identified social interaction and social capital as meaningful mechanisms and outcomes of sport participation and the governance of public sports spaces [40,41]. Research in sport-event settings has further distinguished bonding, bridging, and linking social capital as separate forms of social value generated through sport-related environments [42]. Although the institutional setting differs from everyday public sport facilities, this evidence supports the inclusion of social connectedness as a substantive outcome-side domain.

These three indicators illustrate the value of considering descriptive importance, conditional statistical contribution, measurement specification, and conceptual coverage together. Model A preserves the broader content of the surveyed candidate domain. Models B and C show that the principal structural association remains comparable under a narrower indicator set and alternative specifications. The similarity of the three structural estimates indicates that the observed association was not highly sensitive to the specifications examined. Subsequent multi-source and stakeholder validation can refine the substantive coverage of the indicator domain.

### 5.4. Implications for staged operational performance measurement

The framework supports a staged pathway from indicator design to operational performance measurement. The present study addresses the first stage by specifying a candidate indicator domain and clarifying the conceptual roles of its components. A second stage would operationalise these components using observed data. Provision-side indicators could be linked to administrative staffing records, instructor allocation, public expenditure, facility inventories, and facility-area records. Outcome-side indicators could be linked to user surveys, service quality evidence, population-adjusted provision measures, and spatial accessibility data. A third stage would assess the reliability, comparability, sensitivity, and stakeholder legitimacy of the resulting measurement and aggregation procedures.

These importance ratings and outer weights provide survey-based evidence for framework design. Their translation into operational scoring weights would require validation against observed data, explicit weighting principles, stakeholder consultation, and sensitivity analysis. These additional steps would help determine whether participants’ importance judgments correspond to measurable variation in facility provision, user experience, accessibility, and distributive adequacy.

Any subsequent benchmarking or ranking system would also require transparent decisions concerning data quality, indicator direction, scale transformation, normalisation, aggregation, missing-data treatment, and the interpretation of composite scores. The source and legitimacy of the weights would require particular attention because different stakeholder groups may assign different importance to organisational, experiential, and distributive objectives. Research on comparative sport rankings demonstrates that indicator coverage and weighting rules can materially shape the resulting assessments [13].

At this stage, the framework has diagnostic and deliberative value. Managers, policymakers, users, and evaluators could use its structure to identify which dimensions are currently measured, which remain omitted, which depend on unavailable data, and where stakeholder priorities converge or diverge. In this capacity, the framework provides a design scaffold for developing a transparent and multi-source public sport facility performance measurement system.

### 5.5. Limitations and future research

The study has four main limitations. First, the evidence reflects one stakeholder group recruited through purposive professional channels. Although management personnel possess practical knowledge of facility operation, reporting, and resource deployment, the sample does not represent users, non-users, residents, policymakers, or community organisations. Purposive recruitment also limits statistical generalisation to the wider population of such personnel; accordingly, the findings are sample-specific.

Second, all substantive indicators were rated by the same respondents using the same importance-rating format. Although procedural remedies were applied and common method variance was examined diagnostically, common method bias cannot be ruled out. A general tendency to assign consistently high or low ratings across indicator groups may have contributed to the magnitude of the association between the two composites. Replication using multiple stakeholder groups and independent data sources is needed to determine whether the observed coherence extends beyond same-source participant judgments.

Third, the study was conducted in Fujian Province, China, using a cross-sectional design. Governance arrangements, funding responsibilities, facility-management structures, and service expectations may differ across regions and countries. Cross-sectional importance ratings also provide no temporal evidence about relationships between provision conditions and subsequent user-facing or distributive outcomes. Broader system-level inference will require geographic replication and longitudinal operational data.

Fourth, the surveyed indicator domain and its convergent validation remain incomplete. Model A preserved the full candidate set included in the survey, but these indicators do not exhaust the possible dimensions of public sport facility performance. In addition, redundancy analysis could not be conducted for the formatively and compositely specified blocks because the survey did not include an independent global criterion or alternative measure representing the corresponding domains. The reported outer weights, supplementary outer loadings, and collinearity diagnostics therefore characterise the indicator-level behaviour of these blocks but do not provide independent convergent validation. Future studies should broaden the indicator domain and incorporate appropriately designed global criterion measures or independent operational benchmarks for external validation.

Future research can extend the framework in three connected directions. External validation should examine whether the manager-informed domains correspond to observed facility provision, spatial accessibility, user experience, participation, and public-value outcomes. Stakeholder comparison should assess where the importance judgments of management personnel converge with or diverge from those of users, residents, policymakers, and community organisations. Explanatory extension should examine theoretically specified organisational and contextual predictors, including organisational affiliation, facility type, managerial experience, governance arrangements, reporting requirements, and local policy context. Together, these extensions would move the framework from manager-informed indicator organisation toward multi-source operational performance measurement.

## 6. Conclusion

This study developed a manager-informed input–output indicator framework for organising candidate indicators prior to operational public sport facility performance measurement. Relatively high ratings across both domains and the positive association between their composites indicate alignment between provision-side and outcome-side priorities within the observed judgments.

The study also illustrates the importance of aligning measurement specification with indicator heterogeneity. Model A specified both blocks as composites and retained all 13 surveyed candidate indicators to preserve domain coverage. Similar structural estimates across the two reduced-indicator sensitivity models indicate that the principal association remained stable under narrower indicator coverage and alternative measurement specifications. Within this design, the outer weights describe conditional indicator contributions and can inform the design and subsequent validation of operational weighting approaches.

The framework provides a preparatory structure for organising candidate indicators and guiding their subsequent validation with observed provision and outcome data. Further development will require the integration of administrative, financial, facility, spatial, policy, user, and community evidence, together with comparison across stakeholder groups and institutional settings.

## Supporting information

S1 Appendix Questionnaire used in the survey of public sport facility management personnel. (DOCX)

S1 Data Anonymised survey dataset with codebook. (XLSX)

S1 Table Indicator-level measurement results for sensitivity Models B and C. (DOCX)
