# Supplementary material for: Developing a manager-informed indicator framework for public sport facility performance measurement: Evidence from perceived indicator importance
Source: PLoS One. 2026 Nov 13;21(11):e0361375. doi: 10.1371/journal.pone.0361375 (PMC13766266; doi:10.1371/journal.pone.0361375)
Supplement: Supplementary file 1 [file pone.0361375.s001.docx]

**S1 Appendix. Questionnaire used in the survey of public sport facility management personnel**

This appendix presents the questionnaire items used to collect perceived importance ratings from public sport facility management personnel regarding candidate indicators for inclusion in a future public sport facility performance measurement framework. Respondents rated each candidate indicator on a 5-point Likert-type importance scale ranging from 1 = very unimportant to 5 = very important.

Questionnaire title: Questionnaire on Candidate Indicators for Public Sport Facility Performance Measurement

Hello, and thank you for contributing to this research. This questionnaire seeks your views on the importance of candidate indicators for inclusion in a future input–output framework for public sport facility performance measurement. The target respondents are public sport facility management personnel. Please rate the importance of each candidate indicator.

Section A. Demographic profile

1. Consent
2. Gender
3. Age
4. Education level
5. Workplace affiliation
6. Experience in sports facility development, management, or research

Section B. Candidate indicators

Response scale:

1 = Very unimportant

2 = Unimportant

3 = Neutral

4 = Important

5 = Very important

Input-side indicators

Q1. Number of sports administration staff

Q2. Number of social sports instructors

Q3. Public financial investment in public sport facilities

Q4. Number of public sport facilities

Q5. Public sport facility area

Output-side indicators

Q6. Satisfaction with the use of public sport facilities

Q7. Core product quality of public sport facilities

Q8. Service quality of public sport facilities

Q9. Social network quality of public sport facilities

Q10. Relationship investment quality of public sport facilities

Q11. Number of public sport facilities per capita

Q12. Public sport facility area per capita

Q13. Public sport facility area density
