# Supplementary material for: Developing a manager-informed indicator framework for public sport facility performance measurement: Evidence from perceived indicator importance
Source: PLoS One. 2026 Nov 13;21(11):e0361375. doi: 10.1371/journal.pone.0361375 (PMC13766266; doi:10.1371/journal.pone.0361375)
Supplement: Supplementary file 2 [file pone.0361375.s003.docx]

**S1 Table. Indicator-level measurement results for sensitivity Models B and C**

**Panel A. Model B: formative input-side block**

| **Indicator** | **Outer weight** | **p** | **95% CI** | **Outer loading** | **Loading 95% CI** | **VIF** |
| --- | --- | --- | --- | --- | --- | --- |
| Input 1 | 0.274 | 0.056 | [-0.007, 0.553] | 0.827 | [0.677, 0.927] | 1.977 |
| Input 4 | 0.477 | 0.001 | [0.199, 0.761] | 0.917 | [0.806, 0.979] | 2.215 |
| Input 5 | 0.377 | 0.016 | [0.059, 0.671] | 0.892 | [0.772, 0.962] | 2.308 |

**Panel B. Model B: reflective output-side block**

| **Indicator** | **Outer loading** | **p** | **95% CI** |
| --- | --- | --- | --- |
| Output 1 | 0.812 | < 0.001 | [0.754, 0.861] |
| Output 2 | 0.796 | < 0.001 | [0.732, 0.851] |
| Output 3 | 0.778 | < 0.001 | [0.704, 0.837] |
| Output 5 | 0.814 | < 0.001 | [0.765, 0.855] |
| Output 6 | 0.783 | < 0.001 | [0.725, 0.833] |
| Output 7 | 0.811 | < 0.001 | [0.762, 0.855] |
| Output 8 | 0.775 | < 0.001 | [0.714, 0.828] |

Reflective construct statistics: Cronbach’s α = 0.903; ρ_A = 0.904; composite reliability = 0.924; AVE = 0.633.

**Panel C. Model C: composite input-side and output-side blocks**

| **Indicator** | **Outer weight** | **p** | **95% CI** | **Outer loading** | **Loading 95% CI** | **VIF** |
| --- | --- | --- | --- | --- | --- | --- |
| **Input-side composite** | | | | | | |
| Input 1 | 0.236 | 0.144 | [-0.075, 0.554] | 0.811 | [0.638, 0.927] | 1.977 |
| Input 4 | 0.497 | 0.001 | [0.191, 0.793] | 0.923 | [0.804, 0.983] | 2.215 |
| Input 5 | 0.391 | 0.024 | [0.026, 0.704] | 0.895 | [0.758, 0.968] | 2.308 |
| **Output-side composite** | | | | | | |
| Output 1 | 0.222 | 0.155 | [-0.102, 0.511] | 0.820 | [0.647, 0.909] | 2.189 |
| Output 2 | 0.180 | 0.238 | [-0.112, 0.485] | 0.791 | [0.614, 0.894] | 2.072 |
| Output 3 | 0.185 | 0.162 | [-0.071, 0.448] | 0.765 | [0.584, 0.866] | 2.003 |
| Output 5 | 0.031 | 0.833 | [-0.257, 0.323] | 0.755 | [0.586, 0.854] | 2.333 |
| Output 6 | 0.305 | 0.028 | [0.015, 0.562] | 0.824 | [0.673, 0.907] | 2.057 |
| Output 7 | 0.245 | 0.089 | [-0.035, 0.539] | 0.828 | [0.688, 0.908] | 2.251 |
| Output 8 | 0.078 | 0.586 | [-0.212, 0.353] | 0.725 | [0.541, 0.836] | 2.054 |

**Note**. CI = percentile bootstrap 95% confidence interval based on 5,000 subsamples. Model B specified the input-side block formatively and the output-side block reflectively. Model C specified both reduced indicator blocks as composites. For the formative and composite blocks, outer weights represent indicators’ unique contributions conditional on the other indicators in the same block, whereas outer loadings provide supplementary evidence of absolute indicator relevance. Collinearity was assessed using outer-model variance inflation factors (VIFs). For the reflective output-side construct in Model B, indicator-level assessment was based on outer loadings and bootstrap confidence intervals; construct-level reliability and AVE are reported below Panel B.
